# Numerical Simulation and Optimization of Directional Solidification Process of Single Crystal Superalloy Casting

**DOI:** 10.3390/ma7031625

**Published:** 2014-02-28

**Authors:** Hang Zhang, Qingyan Xu, Baicheng Liu

**Affiliations:** Key Laboratory for Advanced Materials Processing Technology, Ministry of Education, School of Materials Science and Engineering, Tsinghua University, Beijing 100084, China; E-Mails: zhanghangmu@hotmail.com (H.Z.); liubc@tsinghua.edu.cn (B.L.)

**Keywords:** numerical simulation, directional solidification, single crystal superalloy, fuzzy controlling strategy

## Abstract

The rapid development of numerical modeling techniques has led to more accurate results in modeling metal solidification processes. In this study, the cellular automaton-finite difference (CA-FD) method was used to simulate the directional solidification (DS) process of single crystal (SX) superalloy blade samples. Experiments were carried out to validate the simulation results. Meanwhile, an intelligent model based on fuzzy control theory was built to optimize the complicate DS process. Several key parameters, such as mushy zone width and temperature difference at the cast-mold interface, were recognized as the input variables. The input variables were functioned with the multivariable fuzzy rule to get the output adjustment of withdrawal rate (*v*) (a key technological parameter). The multivariable fuzzy rule was built, based on the structure feature of casting, such as the relationship between section area, and the delay time of the temperature change response by changing *v*, and the professional experience of the operator as well. Then, the fuzzy controlling model coupled with CA-FD method could be used to optimize *v* in real-time during the manufacturing process. The optimized process was proven to be more flexible and adaptive for a steady and stray-grain free DS process.

## 1. Introduction

With the rapid development of computer and information technology, computer aided manufacturing technologies (computer-aided design (CAD), computer aided engineering (CAE), computer-aided manufacturing (CAM), *etc.*) are widely used in industrial production [1–10]. The numerical simulation method used in the directional solidification (DS) process of superalloys is receiving more attention in aviation and energy industries [11–18].

An important application of the numerical simulation is to predict casting defects, such as shrinkage cavity, hot cracking, and single crystal integrity during the directional solidification of the turbine blades [19–24]. The integrity of single crystal is a major index for the production of single crystal (SX) blades. Stray grain is a usual defect, which is a focal point of simulation studies [23–28].

The DS process, with many controlling parameters, is a complicated process. There are interactive effects among these parameters, which leads to a very narrow process window for the manufacturing of SX blades. Withdrawal rate is one of the important parameters influencing the DS process. A great deal of research has been done to study the relationship between withdrawal rate, microstructure, and properties [25,29–33]. A constant withdrawal rate is often used in industries because it is easy and convenient to control, however, it has less flexibility and leads to a low yield rate of SX blades. The experiment-based variable withdrawal rate for DS process develops quickly and is adopted more and more [34,35]. However, the main problem is that it needs many trials, increases cost, and enlarges time circles.

The numerical simulation used in DS process provides an effective way to lower the experimental circle and cost. In addition, some simple variable withdrawal rate processes were already proposed by simulation technology for an efficient and defect-free DS process [29,34,35]. However, these studies are still limited to modeling the experiment with a few times of rate changing. The process improvement lacked guidelines, which still meant a method of trial and error, and did not make full use of numerical simulation techniques. There are some studies [36,37] on the optimizing of DS processes, based on modeling and simulation, however, new models and algorithms need to be developed to deal with the system’s variables, to instantly adjust the solidification parameters, and, finally, to improve the DS process.

The directional solidification is a complicate nonlinear system and hard to describe using a precise mathematical model. Fuzzy controlling method, based on fuzzy set theory [38], can deal with the fuzzy relation of semantic variables easily, according to certain fuzzy rules. It is an expert in nonlinear, close coupling, and uncertain systems [39–43]. In this work, the fuzzy controlling model was built to optimize the directional solidification process. The interface temperature gradient and average mushy zone width were studied in detail. Through optimizing the withdrawal rate instantly during the calculation of the directional solidification process, the fuzzy controlling model aims to get a higher temperature gradient and improve the stability of the directional solidification process.

## 2. Physical and Mathematical Models

### 2.1. Directional Solidification Process

**Figure 1.**
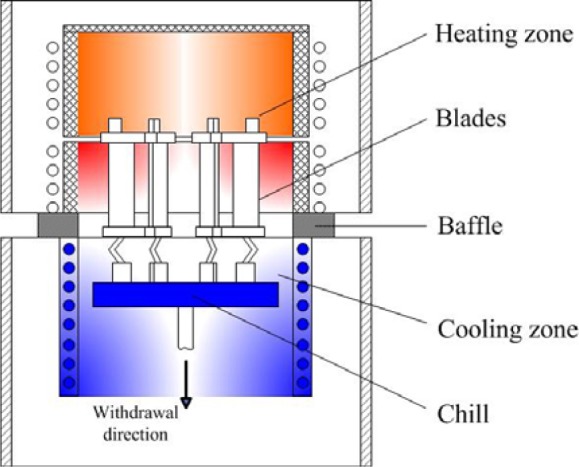
Schematics of a Bridgman furnace.

There are different pieces of equipment used in DS processes, and the Bridgeman furnace is one of the most widely used. This furnace can be simplified and divided into five parts for modeling and simulation: heating zone, Baffler, Cooling zone, Chill, and Withdrawal unit, as shown in Figure 1. If the Cooling zone is equipped with water-cooled copper rings, the Bridgeman furnace is used for the high rapid solidification (HRS) technology, which is a main DS method to produce superalloy blade castings.

In the HRS DS process, a group of mold shells are fixed on the chill. The liquid metal is poured into the mold and kept for minutes to make the temperature high enough. Then, the withdrawal unit starts, at certain speeds, of which the value is constant or variable. The baffler isolates the heating zone and cooling zone, then a unidirectional temperature gradient forms. When the liquid metal is drawn to pass the baffler, or entirely into the cooling zone, the mushy zone begins to freeze.

### 2.2. The Scheme of Optimizing DS Processes by Simulation Technology

**Figure 2.**
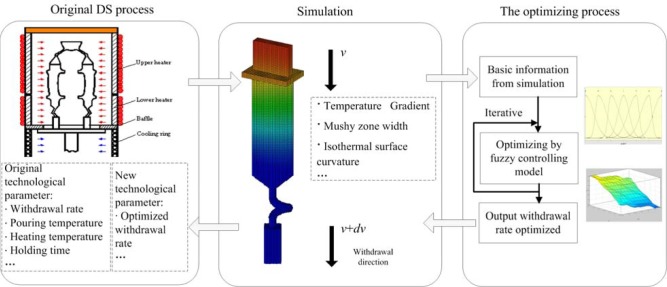
The framework of DS processes, optimized by simulation.

**Figure 3.**
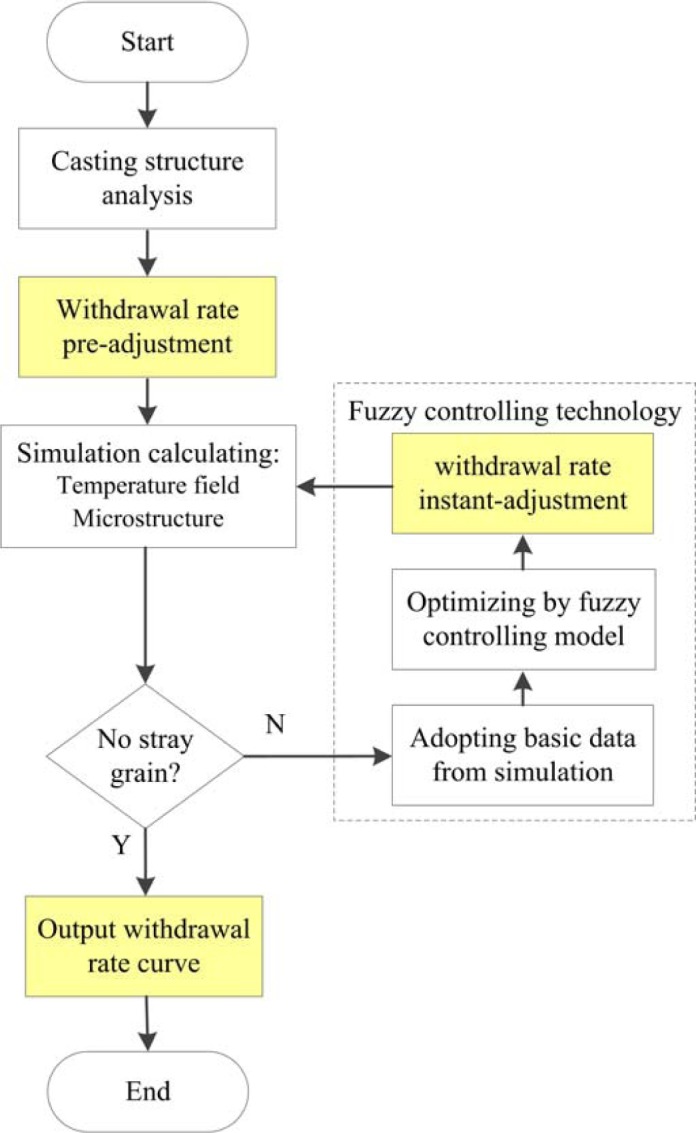
The flow chart of withdrawal rate optimizing by simulation.

Simulation technology was used to simulate DS processes for years. In this study, the fuzzy controlling model was built to optimize the withdrawal rate by simulation technology, as shown in Figures 2 and 3.

Firstly, the casting model was input and the withdrawal rate was pre-adjusted, based on the shape of the input model. Then, the DS process was simulated by the CA-FD method. The temperature field and microstructure growth were calculated step by step. Some key parameters, such as temperature gradient and mushy zone width, were analyzed by the fuzzy controlling model. The withdrawal rate was adjusted instantly. Then the optimized withdrawal rate was changed in the new simulation. During the simulating process, if there were stray grains appeared on the casting, the withdrawal rate would be post-adjusted and the simulating process would feed back to calculate again with a new withdrawal rate. Finally, when there is no defects predicted in the calculation, a withdrawal rate curve will be given out, which would be more effective and stable for the real DS process.

### 2.3. The Fuzzy Control Model for Optimizing DS Processes

A fuzzy control model was built to analyze the real time data derived from the simulation process, and some key variables were tracked to adjust the withdrawal rate.

#### 2.3.1. The Fuzzy Controller

**Figure 4.**
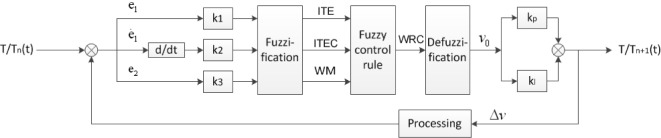
The fuzzy controlling system.

The mushy zone of DS process was studied by adopting a single output and three inputs model, as shown in Figure 4.

The three input variables are follows: ITE (the casting-mold interface temperature error) is the temperature difference between most inner cell’s temperature in shell and the most outer cell’s temperature in cast; ITEC is the change of ITE; WM is the average width of mushy zone. The output variable is SC (withdrawal speed change).

#### 2.3.2. The Domain of Discourse of Fuzzy Variables and Membership Functions

ITE was quantization into 13 grades, which are {−6, −5, −4, −3, −2, −1, +0, +1, +2, +3, +4, +5, +6}. The fuzzy subsets are {PB, PM, PS, O, NS, NM, NB}. e_1_, ė_1_ and e_2_ are the accurate values of ITE, ITEC, and WM, respectively, and the corresponding quantification factors are $k_{e_{\text{1}}}$, ${\text{k}}_{\dot{e}}{}_{{}_{1}}$, and ${\text{k}}_{\text{e}}{}_{{}_{2}}$. The membership function of ITE is shown as Equation (1). The other fuzzy variables, such as ITEC, WM, and SC, are similarly treated with ITE. In the equation, *a*, *b*, and *c* are the parameters of the membership function.

$$f_{\text{ITE}(x)}=\{\begin{array}{c} \text{exp}(-\frac{{\left(x-a\right)}^{2}}{2b^{2}}),\;x\leq a\\ \text{exp}(-\frac{{\left(x-a\right)}^{2}}{2c^{2}}),\;x\geq a \end{array}$$ (1)

#### 2.3.3. Fuzzy Rules for Withdrawal Rate Adjustment

Fuzzy rules could be described as follows: if WM is WM*_i_* and ITE is ITE*_j_*, and ITEC is ITEC*_k_*, SC is SC*_ijk_*.

The fuzzy relation can be written as Equations (2) and (3):

$$R_{ijk}=({\text{WM}}_{i}\times {\text{SC}}_{ijk})\wedge ({\text{ITE}}_{j}\times {\text{SC}}_{ijk})\wedge ({\text{ITEC}}_{k}\times {\text{SC}}_{ijk})=({\text{WM}}_{i}\times {\text{ITE}}_{j}\times {\text{ITEC}}_{k})\times {\text{SC}}_{ijk}$$ (2)

$$R=R_{111}+R_{112}+\cdots +R_{ijk}+\cdots +R_{opq}=\underset{i,j,k}{\cup }R_{ijk}\;i=1,2,\ldots ,o\;j=1,2,\ldots ,p\;k=1,2,\ldots ,q$$ (3)

where WM*_i_*, ITE*_j_*, ITEC*_k_*, and SC*_ijk_* are the semantic input variables of WM, ITE, ITEC, and WRC, respectively.

During the simulation calculation of DS process, e_1_, ė_1_, and e_2_ were received, as well as the semantic input variables, such as WM*_i_*, ITE*_j_*, and ITEC*_k_*. The semantic output variable of fuzzy controller is calculated based on Equation (4):

$$\begin{array}{c} {\text{SC}}^{\delta }=({\text{WM}}_{r}\times {\text{ITE}}_{s}\times {\text{ITEC}}_{t})○R=({\text{WM}}_{r}\times {\text{ITE}}_{s}\times {\text{ITEC}}_{t})○\underset{i,j,k}{\cup }R_{ijk}\\ =\underset{i,j,k}{\cup }({\text{WM}}_{r}\times {\text{ITE}}_{s}\times {\text{ITEC}}_{t})○({\text{WM}}_{i}\times {\text{ITE}}_{j}\times {\text{ITEC}}_{k})\times {\text{SC}}_{ijk}\\ =\underset{i,j,k}{\cup }\text{sup}[({\text{WM}}_{r}\times {\text{ITE}}_{s}\times {\text{ITEC}}_{t})\wedge ({\text{WM}}_{i}\times {\text{ITE}}_{j}\times {\text{ITEC}}_{k})]\wedge {\text{SC}}_{ijk} \end{array}$$ (4)

*v*(*t*) can be calculated based on the defuzzification of _SC_^δ^, shown as Equation (5):

$$v(t)=\Delta v+v(t-1)=ke\cdot sgn({\text{SC}}^{\delta })\cdot Int({\text{SC}}^{\delta }+0.5)+v(t-1)$$ (5)

*k_e_* is the scale factor. sgn(*x*) is sign function. Int(*x*) is rounding function.

## 3. The Optimizing Process by Simulation

### 3.1. The Basic Simulation Condition

The SX sample blade designed has the main features of a real SX blade: the whole length is over 200 mm; the platform has an abrupt change of section; the body of the blade rotates at a certain angle respective to the tenon. The experimental material was Ni-based superalloy DD6 [44,45]. There were four schemes for simulation: group SG1 with the rate of 7.0 mm/min, group SG2 with the rate of 4.5 mm/min, group SG3 with the rate of 1.0 mm/min and group SG4 with the rate optimized by fuzzy controlling model. Two groups were selected for experimental study: Group EG1 with the rate of 7.0 mm/min and Group EG2 with the rate of 4.5 mm/min. The parameters used in these simulating groups are shown as Table 1.

### 3.2. The Optimized Variable Withdrawal Rate Process

**Figure 5.**
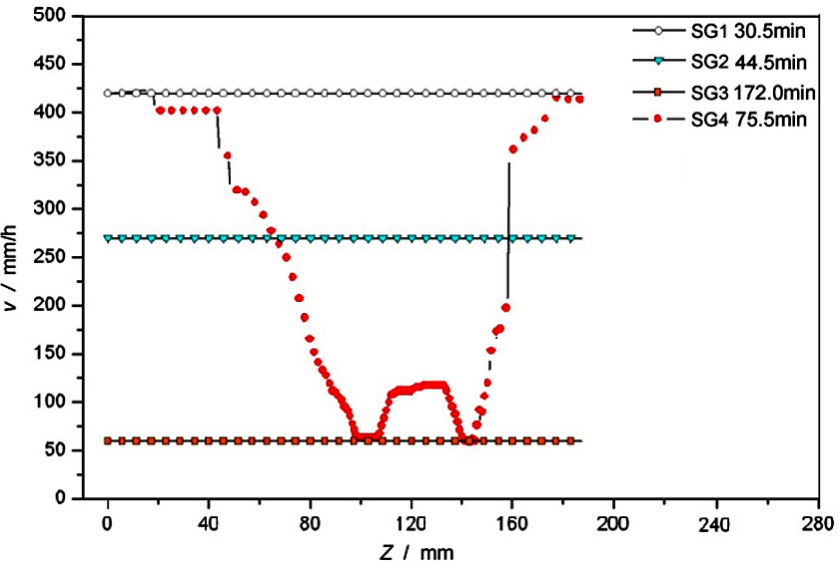
Curves of withdrawal rates of SG1–SG4.

The optimized withdrawal rate of DS process was obtained in the group SG4. Figure 5 shows the curves of withdrawal rates of groups SG1–SG4. From the simulation results, it can be seen that the solidification time of SG4 was 75.5 min, which was half that of SG3, but longer than that of SG2.

**Figure 6.**
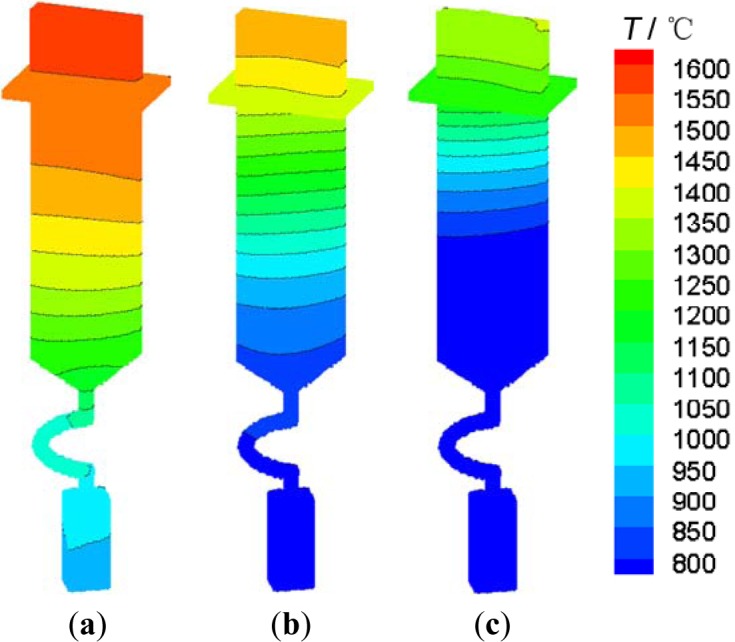
Temperature distribution of SG4 at different time (**a**) 19 min; (**b**) 27.5 min; (**c**) 36 min.

The solidification processes of the four groups of DS were calculated. Then, the temperature distribution, mushy zones and temperature gradients of different processes could be analyzed by solidification times. Figure 6 is the temperature distributions of SG4 during the DS process. These temperature cloud charts show the unidirectional heat diffusion and temperature gradient distribution. The isothermal lines basically kept horizontal and some showed a slight slant, or concave or convex.

### 3.3. The Comparison of Constant and Optimized Withdrawal Rates Processes

**Figure 7.**
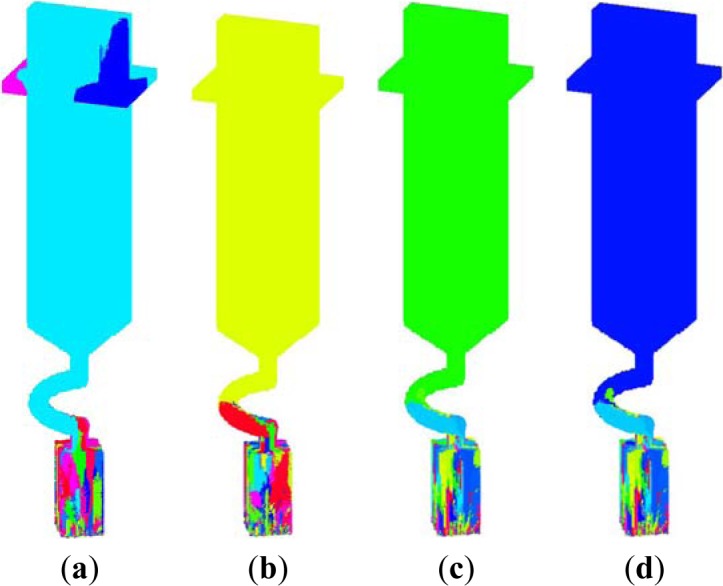
Simulated Microstructures of the four groups (**a**) SG1; (**b**) SG2; (**c**) SG3; (**d**) SG4.

Figure 7 is the microstructures of SG1–SG4 by simulation. SG1 with a constant rate of 7 mm/min appeared some stray grains in the platform, contrast to other groups. These stray grains disqualified the casting, which had different orientations from the single grain grown from the blade body. In Figure 7, although there were no stray grains at the other blades, SG2 had a very narrow process window and would tend to form stray grains at small fluctuations of other process parameters, and SG3 was of too low efficiency and had too many defects to be applied in the industry production, which will be further explained below. Thus, SG4 could have a higher productivity and no stray grains.

**Figure 8.**
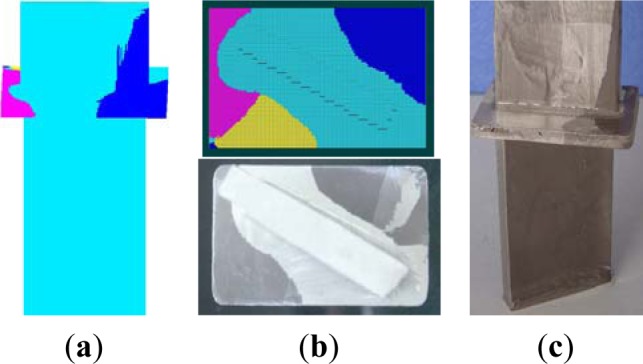
The comparison of microstructure of SG1with EG1 (**a**) The simualtion of microstructure of SX blade (partly); (**b**) The comparison between simulation and experiment (upward view, *i.e.*, the bottom surface of the platform); (**c**)The experimental result of EG1.

Stray grain is one of most severe defects of SX blade. Figure 8 is the comparison of microstructure of EG1 and SG1. Both results of EG1 and SG1 showed that the stray grains mostly started and formed in the platform, where were far from the root of the tenon and at a deep overcooling, as shown in Figure 8a,c. From Figure 8b, the locations and boundaries of stray grains predicted by simulation were similarly with that by experiment. And it’s proved that the simulating models for heat transfer and microstructure evolution were accurate to predict the DS process of SX blade well.

There are many reasons which caused stray grains, such as impurity of the melt, ceramic sunken surface, the surface of metal needles for fastening position, as well as the formation of deep undercooling zone. The deep undercooling will be the main factor leading to the stray grains in the real SX turbine blades.

**Figure 9.**
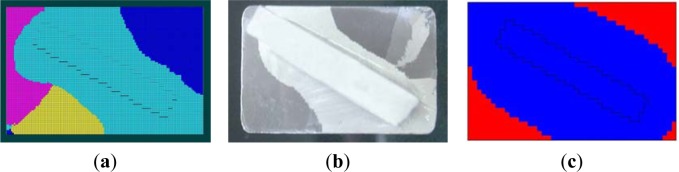
The comparison beween the simulation and experiment to show stray grains formations (upward view, *i.e.*, the bottom surface of the platform) (**a**) simulation result (SG1); (**b**) experiment result (EG1); (**c**) isolated undercooling zones (IUZ) of SG1.

In this study, SG1 and EG1 show obvious stray grains at the platform, as shown in Figure 9a,b. The undercooling zone was the main area that a stray grain nucleated and grew, which was caused by the non-uniform temperature distribution. As for SG1 and EG1, the stray grains formed at platforms. In Figure 9c, the special zones were marked in the simulated results, where the cells did not solidify but of which temperatures were lower than liquidus. These cells were named isolated undercooling zones (IUZs). IUZ provided the low temperature melt for stray grain nucleation and growth. IUZ was formed on certain condition of DS process, for example, a faster withdrawal rate.

According to the definition above, IUZ has some features: (a) Stray grains of SX blades are mainly formed in IUZs; (b) The location and range of an IUZ are influenced and divided based on the temperature distribution; (c) IUZ often appears at some tips of the casting where heat dissipation is faster; (d) The analysis of IUZ is a convenient way to predict areas of the stray grain formation.

**Figure 10.**
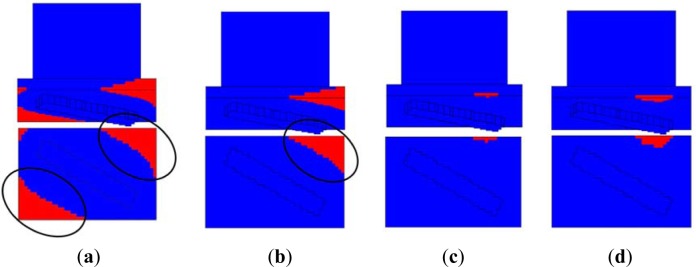
The IUZ distributions of the four simulation groups (**a**) SG1; (**b**) SG2; (**c**) SG3; (**d**) SG4. The upper figures are side views in 3D, and the lower figures are the vertical views of the platform.

Figure 10 shows the IUZs formed during the calculations of SG1–SG4. The IUZs of SG1 were larger in range and more in number, as shown in Figure 10a. IUZs of SG3 and SG4 were smaller than others, which was the main reason for no stray grains formed in these groups. In addition, IUZs of SG2 were inclined to formed stray grains, but at the critical status.

There are lots of factors influenced on the formation of IUZ, and withdrawal rate is an important one. The quick change of the cross-section area at the platform allowed more heat dissipation from the part below the platform, such as cooling zone or the water-cooled copper plate. Then at some edge or corner of the platform, the melt has a lower temperature than liquidus, but not turned to be solid, and the IUZs were formed.

Based on the analysis above, a smaller IUZ is expected by adjusting the withdrawal rate. A lower withdrawal rate is benefit for keeping the IUZs at the heating zone of the furnace as long as possible. Then, the heat radiation would warm up these IUZs and compress their regions. Then, stray grain has no time and space to nucleate and grow. It provides a method for the fuzzy controlling model to optimize withdrawal rate dynamically, as shown in Figure 10d.

## 4. Advantages of the Fuzzy Optimizing Model

### 4.1. To Get Higher Temperature Gradient

**Figure 11.**
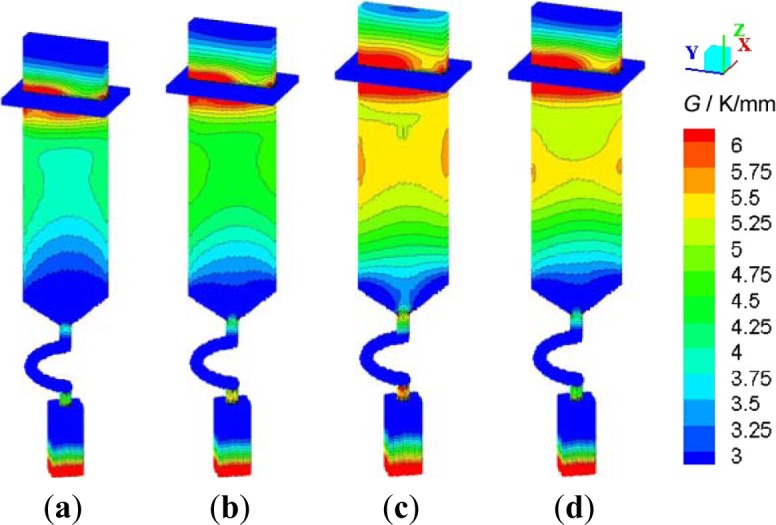
Temperature gradients of mushy zones during DS process (**a**) SG1; (**b**) SG2; (**c**) SG3; (**d**) SG4.

Temperature gradient of mushy zone is a major parameter of the DS process, which influences the grain growth and quality of final castings. In the study, temperature gradients in mushy zones during solidification process were sampled (interval: 0.5 min), as shown in Figure 11. In all four groups, temperature gradients at bottom of starter block and at center of platform were higher than 6 K/mm, which resulted from the fast heat transfer of water-cooled chill plate and the sudden change of section area at the platform, respectively. Meanwhile, temperature gradients during the whole solidification process of SG3 and SG4 were higher than that of SG1 and SG2, which showed the advantages of these two processes.

In conclusion, SG4 had a short solidification time than SG3, as shown in Figure 5, but its temperature gradients were higher than others. That is to say, at a proper time span, the DS process with an optimized withdrawal rate is benefit for improving microstructure and properties, and increasing productivity and yield rate of SX blades.

### 4.2. To Enlarge the Process Window

DS process has many controlling parameters, which are complicate and coupled closely. Thus, the process is narrow for DS casting, which means a minor fluctuation may be amplified and lead to the formation of stray grains or other defects.

**Figure 12.**
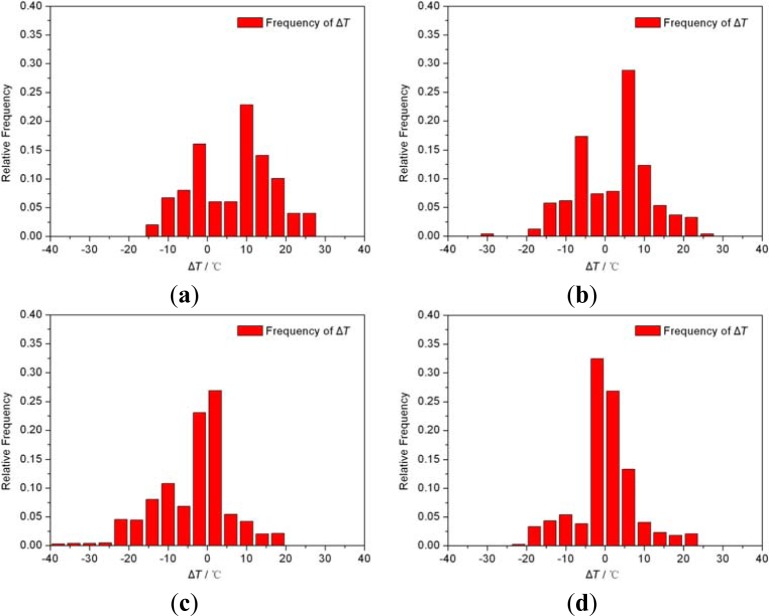
The frequency distributions of Δ*T* during the solidification time (**a**) SG1; (**b**) SG2; (**c**) SG3; (**d**) SG4.

In the work, the frequencies of Δ*T* (the physical value of ITE) and mushy zone width were studied during the whole solidification process, and the sampling interval is 30 s. Figure 12 shows the frequencies of Δ*T*_s_ during the solidification process of the four groups. When the Δ*T* is zero, it means the transverse temperature gradient is eliminated, which is ideal for a DS process. In Figure 12d, most of Δ*T*_s_ were assembling around zero, which means the optimized process could keep a good unidirectional heat flux. Particularly noteworthy is that most frequencies of Δ*T*_s_ in Figure 12c were not good as those in Figure 12d. However, in Figure 12a,b, most frequencies of Δ*T*_s_ correspond to around 5 °C or more. The SG4 had a relative longer time when Δ*T*_s_ equaled to 0, which means a better DS condition than others.

**Figure 13.**
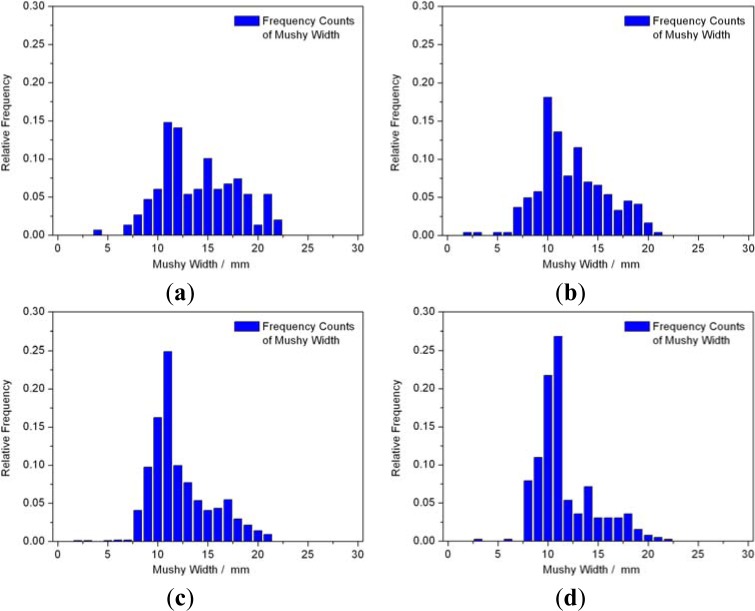
The frequency distributions of mushy zone width of the four simulation groups (**a**) SG1; (**b**) SG2; (**c**) SG3; (**d**) SG4.

Figure 13 shows the frequency distributions of WM of the four simulation groups. The frequency distributions of SG1 and SG2 were basically uniform and approximately distributed at the range of 7.5–20 mm. Most frequencies of WMs of SG4 concentrated around 10 mm and the frequency rate was above 0.25, better than that of SG3, as shown in Figure 13c. This concentration made sure the stabilities of WM and temperature gradient of mushy zone, which was benefit for a more stable DS process and allowed other solidification parameters to adjust in a lager range.

## 5. Conclusions

The mathematical and physical models were built for the DS process. The SX blade was studied by numerical simulation and experimental methods. Stray grain formations were successfully predicted by simulation, and the IUZ was proposed to analyze the mechanism of stray grain formation at the platform.

A fuzzy controlling model for optimizing the DS withdrawal rate was built, and the withdrawal rate for SX blade casting was optimized. The optimized process could successfully get sound SX blade castings.

The advantages of optimized process were analyzed, based on the comparisons of temperature gradients, ITG, and WM. The proposed optimized technology in the paper that couples the intelligent controlling technology and the simulation technology is a useful way to optimize the DS process withdrawal rate and the model is useful to get a higher temperature gradient and enlarge the DS process window.

## Figures and Tables

**Table 1.** The parameters used in the calculation (Superalloy DD6 [44,45]).

Parameters	Unit	Value
Liquidus	°C	1370
Solidus	°C	1310
Density of alloy	g/cm^3^	8.78
Density of shell	g/cm^3^	2.50
Temperature of cooling water	°C	25
